# Mindful attention awareness and attitudes toward social handwashing among nursing and midwifery students

**DOI:** 10.1590/1980-220X-REEUSP-2025-0095en

**Published:** 2025-10-03

**Authors:** Leyla Baran, Huri Öztürk, Feride Doğru Mungan

**Affiliations:** 1 Mardin Artuklu University, Faculty of Health Sciences, Nursing Department, Mardin, Türkiye.; 2 Izmir Democracy University, Vocational School of Health Services, Elderly Care Department, İzmir, Türkiye.; 3 Mardin Artuklu University, The Institute for Graduate Educational Studies, Health Administration, Mardin, Türkiye.

**Keywords:** Hand Disinfection, Mindfulness, Students, Nursing, Desinfecção das Mãos, Atenção Plena, Estudantes de Enfermagem

## Abstract

**Objective:**

To explore the relationship between mindful attention awareness and attitudes toward social handwashing among undergraduate nursing and midwifery students.

**Method:**

A cross–sectional and correlational study was conducted with 679 undergraduate nursing and midwifery students. Data were collected using two scales: (1) the Mindful Attention Awareness Scale (MAAS) and (2) the Attitude Scale toward Social Handwashing (ASSHW). Additionally, the participants provided data on their sociodemographic characteristics and responded to an inventory of handwashing practices. Relationships between scores were examined using Spearman’s rho.

**Results:**

The students’ mindful attention levels (MAAS) averaged 44.97 ± 11.46, and their attitudes toward social handwashing (ASSHW) averaged 78.32 ± 13.67. While no direct correlation was found between overall ASSHW and MAAS scores, there were positive correlations between mindful attention levels and certain ASSHW sub-dimensions, as well as with specific handwashing behaviors such as frequency and duration.

**Conclusion:**

These findings suggest that mindful attention may support better hand hygiene practices among nursing and midwifery students.

## RESUMO

**Objetivo:**

Investigar a relação entre a consciência plena da atenção e as atitudes em relação à lavagem social das mãos entre estudantes de graduação em enfermagem e obstetrícia.

**Método:**

Estudo transversal e correlacional realizado com 679 estudantes de graduação em enfermagem e obstetrícia. Os dados foram coletados por meio de duas escalas: (1) a Escala de Consciência Plena da Atenção (ECPA) e (2) a Escala de Atitudes em Relação à Lavagem Social das Mãos (EARLSM). Além disso, os participantes forneceram informações sociodemográficas e preencheram um inventário de práticas de lavagem das mãos. As relações entre os escores foram analisadas utilizando o coeficiente de correlação de Spearman.

**Resultados:**

Os níveis de atenção plena (ECPA) apresentaram média de 44.97 ± 11.46, e as atitudes em relação à lavagem social das mãos (EARLSM) apresentaram média de 78.32 ± 13.67. Embora não tenha sido observada correlação direta entre os escores totais das escalas ECPA e EARLSM, foram encontradas correlações positivas entre os níveis de atenção plena e determinadas subdimensões da EARLSM, bem como com comportamentos específicos de lavagem das mãos, como frequência e duração.

**Conclusão:**

Esses achados sugerem que a consciência plena pode favorecer melhores práticas de higiene das mãos entre estudantes de enfermagem e obstetrícia.

## INTRODUCTION

Mindfulness involves intentionally focusing on the present moment with an open and unbiased attitude^(1)^. Its function is to prevent individuals from acting on autopilot -reacting based on automatic, unexamined thoughts-and instead foster a conscious awareness of unfolding experiences^(2)^. This heightened awareness enables individuals to pause, observe, and choose intentional responses rather than habitual reactions^(3)^.

Extensive research has shown that mindful attention plays a protective role in mental health: it buffers stress^(4)^, supports coping with anxiety and depression^(5)^, and contributes to overall well-being^(6)^. Beyond psychological benefits, mindful attention has also been linked to better adherence to preventive health measures -such as social distancing- during the COVID-19 pandemic^(7)^, suggesting that mindful individuals are more likely to engage in behaviors driven by conscious intention. However, this association has not always extended to handwashing, a critical yet often automated hygiene practice^(7,8)^. Hand hygiene is performed frequently in clinical settings -especially by nursing and midwifery professionals- and is prone to lapses when attention is absent^(9)^. Yet, it offers a prime opportunity to integrate mindfulness: by refocusing attention during routine handwashing, individuals can embed a moment of intentional awareness into their daily practice^(2)^.

From a theoretical standpoint, mindfulness encourages intentional behavior by disrupting automaticity and enhancing metacognitive awareness. When applied to hand hygiene, this means that mindful attention awareness may increase the likelihood of performing the behavior reliably, conscientiously, and reflectively. Moreover, nursing theory frames hand hygiene as an act of self-care that shapes workplace culture -highlighting the potential of mindfulness interventions to strengthen both personal habits and broader organizational norms^(8)^.

Therefore, this study aimed to investigate the relationship between mindful awareness and attitudes toward handwashing among nursing and midwifery students. Comparative analyses were also conducted to explore differences in mindful awareness and hand hygiene behaviors (frequency and duration) across gender, academic program, and accommodation status. We hypothesized:

H_1_:
Nursing and midwifery students have positive attitudes toward social handwashing.
H_2_:
Nursing and midwifery students demonstrate high levels of mindful attention and awareness.
H_3_:
Nursing and midwifery students’ levels of mindful ­attention and awareness are associated with their social handwashing attitudes.

## METHOD

### Design of the Study

In line with quantitative research principles that explore associations between variables or sets of scores, this study used a cross-sectional correlational research design to examine the relationship between hand hygiene behavior and mindful attention awareness^(10)^.

### Population

All nursing and midwifery students ranging from first-year to graduate level were recruited from the Faculty of Healthcare Sciences at Mardin Artuklu University during the 2023–2024 academic year.

### Selection Criteria

Instead of pre-determining a sample size, a simple random sampling method was employed to include data from the entire target population. Eligibility criteria included being enrolled in the nursing and midwifery degree programs during the academic year and understanding and voluntarily completing the data collection forms. Students who did not sign the informed consent for participation were excluded.

### Sample Definition

The total population of nursing and midwifery students at the university was 771 (417 nursing students and 354 midwifery students). Of these, ten students were absent when data were collected, and 82 declined to participate, resulting in a response rate of 88%. Hence, 679 students were involved in the study.

### Data Collection

***Demographic Characteristics Questionnaire:*** The demographic questionnaire was developed by the researchers based on the recent literature. It included items such as age, gender, accomodation type, and questions related to the participants’ handwashing habits.

***The Attitude Scale towards Social HandWashing on Nursing Students (ASSHW):*** The ASSHW evaluates nursing students’ attitudes toward handwashing^(11)^. Permission to use the scale was obtained from its authors.

The scale consisted of 20 items, with 9 positively phrased and 11 negatively phrased statements responded on a 5-point Likert format. Scores obtained from the scale range from 20 to 100, where higher scores indicate more positive attitudes toward handwashing. The scale sub-dimensions and corresponding score ranges are as follows:

- The first sub-dimension, Impact of Work Intensity (IWI), includes items 1-11, with possible scores ranging from 11 (lowest) to 55 (highest).
- The second sub-dimension, Impact of Handwashing on Development of Infection (IHDI), includes items 12 to 16, with possible scores ranging from 5 (lowest) to 25 (highest).
- The third sub-dimension, Desire to Learn Handwashing (DLH), includes items 17 to 20, with possible scores ranging from 4 (lowest) to 20 (highest).

***The Mindful Attention Awareness Scale (MAAS):*** The MAAS assesses individual differences in mindfulness by measuring awareness and attention to present experiences in daily life^(12)^. It consists of 15 items rated on a 6-point Likert scale (from “almost always” to “almost never”). Higher scores indicate greater mindfulness. MAAS has a single-factor structure, yielding a single total score. The internal consistency of the scale was reported as 0.82. The Turkish adaptation demonstrated item factor loadings between 0.48 and 0.81, a Cronbach’s alpha reliability of 0.80, and a test–retest reliability of 0.86^(13)^. Permission to use the Turkish version of the scale was granted.

***Hand Hygiene Practice Inventory (HHP):*** The HHP is a self-report questionnaire designed to assess when students perceive a need for handwashing. It consists of brief items rated on a 5-point Likert scale.

### Data Analyses

Data were analysed using the Statistical Package for the Social Sciences (SPSS) version 26.0. Descriptive statistics (means, standard deviations, and percentages) were used to summarize demographic characteristics and scale scores.

The normality of the data was assessed using the Kolmogorov-Smirnov and Shapiro-Wilk tests. Results indicated that all variables, including daily handwashing frequency, handwashing duration, and scale scores (ASSHW, IWI, IHDI, DLH, MAAS, HHPI), significantly deviated from normal distribution (p < 0.001). Therefore, non-parametric statistical tests were employed for group comparisons and correlation analyses.

Associations among the scales -as well as between scale scores and handwashing frequency and duration- were examined using Spearman’s rho correlation coefficient. A 95% confidence interval was used, and the significance level was set at *p* < 0.05 for all analyses.

### Ethical Aspects

Approval for this study was granted by the Research Ethics Committee (reference number: 2024/5-14). All procedures adhered to the principles outlined in the Helsinki Declaration. Prior to commencing data collection, the purpose of the study was explained to all participants, and written informed consent was obtained.

## RESULTS

### Descriptive Data

The mean age of participants was 21.78 ± 1.84 years (range 18–30 years). Most students were female (85.9%), more than half (51.25%) were nursing students, 29.6% were fourth-year students, and 75% resided in dormitories (Table 1).

**Table 1:** Sociodemographic features of the participants- Mardin, Türkiye, 2024.

Sociodemographic features		Nursing		Midwifery		Total	
		n	%	n	%	n	%
**Gender**	**Female**	252	72.4	331	100.0	583	85.9
	**Male**	96	27.6	–––	–––	96	14.1
**Year of Study**	**1st year**	71	20.4	68	20.5	139	20.5
	**2nd year**	84	24.1	75	22.7	159	23.4
	**3rd year**	91	26.1	89	26.9	180	26.5
	**4th year**	102	29.3	99	29.9	201	29.6
**Living Status**	**Family home**	53	15.2	43	13.0	96	14.1
	**Relatives Home**	4	1.1	2	0.6	6	0.9
	**Dormitory**	236	67.8	273	82.5	509	75.0
	**Student House**	55	15.8	13	3.9	68	10.0
**Total**		**348**	**100.0**	**331**	**100.0**	**679**	**100**

As shown in Table 2, the average handwashing frequency was 12.70 ± 7.77 times/day (range: 1–50). The participants most commonly washed hands whenever they felt their hands dirty (89.2%) and preferred using water and liquid soap (90.9%) for hand hygiene. The average handwashing duration was reported as 34.59 ± 21.14 seconds (range: 10–120 seconds).

**Table 2:** Distribution of students’ handwashing habits– Mardin, Türkiye, 2024.

Students’ handwashing habits	Nursing		Midwifery		Total	
	n	%	n	%	n	%
**Situations in which students feel the need to wash their hands**						
Before using the restroom	186	53.4	161	48.6	347	51.1
After using the restroom	287	82.5	289	87.3	576	84.8
After returning home from outside	274	78.7	259	78.2	533	78.5
Before eating	265	76.1	232	70.1	497	73.2
After eating	271	77.9	263	79.5	534	78.6
Whenever my hands feel dirty	316	90.8	290	87.6	606	89.2
Before hospital practices	214	61.5	193	58.3	407	59.9
After hospital practices	280	80.5	268	81.0	548	80.7
**Materials Preferred by Students for Hand Hygiene**						
Only water	37	10.6	19	5.7	56	8.2
Water and bar soap	98	28.2	87	26.3	185	27.2
Water and liquid soap	316	90.8	301	90.9	617	90.9
Alcohol–based antiseptic	141	40.5	103	31.1	244	35.9
**Frequency of Forgetting to Wash Hands During the Day**						
Always	16	4.6	14	4.2	30	4.4
Often	14	4.0	22	6.6	36	5.3
Sometimes	75	21.6	57	17.2	132	19.4
Rarely	170	48.9	156	47.1	326	48.0
Never	73	21.0	82	24.8	155	22.8
**Situations affecting students’ handwashing frequency**						
Spending the day at home/outside	288	82.8	262	79.2	550	81.0
Participating in hospital practice	276	79.3	239	72.2	515	75.8
Experiencing a health issue	192	55.2	183	55.3	375	55.2
Being in crowded environments	223	64.1	214	64.7	437	64.4
Performing mechanically dirty tasks	226	64.9	209	63.1	435	64.1
Using public transportation	235	67.5	210	63.4	445	65.5
**Situations preventing students’ handwashing during the day**						
Lack of access to hygiene products	137	39.4	131	39.6	268	39.5
Skin irritation, cracks, or allergic reactions caused by handwashing agents	109	31.3	102	30.8	211	31.1
Doubt about the cleanliness of handwashing products	108	31.0	96	29.0	204	30.0
Inadequate handwashing facilities	177	50.9	162	48.9	339	49.9
Absence of paper towels	95	27.3	78	23.6	173	25.5
Busy schedules or lack of time	112	32.2	117	35.3	229	33.7
Preference for using antiseptic solutions instead of washing	47	13.5	38	11.5	85	12.5
**Students’ opinions on using proper handwashing techniques**						
Yes	91	26.1	100	30.2	191	28.1
Partially	205	58.9	196	59.2	401	59.1
No	37	10.6	28	8.5	65	9.6
No opinion	15	4.3	7	2.1	22	3.2

The most influential factors affecting handwashing frequency were spending time at home or outside (81.0%) and participation in hospital practice (75.8%). Changes in handwashing duration were primarily influenced by the degree of hand dirtiness (47.1%) and urgency (22.2%). The main barriers to handwashing included inadequate hygiene facilities (49.9%), lack of hygiene products (39.5%), and skin irritation caused by cleansing agents (31.1%).

Most students (59.1%) reported partial adherence to proper handwashing techniques, while 28.1% expressed confidence in their technique.

An analysis of the students’ distribution of the scale scores (Table 3) indicated that the mean scale scores were 78.32 ± 13.67 for ASSHW, 42.93 ± 8.61 for IWI, 20.31 ± 5.42 for IHDI, 15.08 ± 4.01 for DLH, and 60.60 ± 12.91 for HHPI, all of which were above the moderate level. The mean MAAS score was 44.97 ± 11.46 for both groups of students.

**Table 3:** Comparative analysis of ASSHW, MAAS, and HHP - Mardin, Türkiye, 2024.

Scales	Item numbers	Nursing		Midwifery		Total	
		Mean ± SD	Min – Max	Mean ± SD	Min – Max	Mean ± SD	Min – Max
**ASSHW**	20	78.19 ± 13.67	35 – 100	78.45 ± 13.68	45 – 100	78.32 ± 13.67	35 – 100
**IWI**	11	43.05 ± 8.69	11 – 55	42.80 ± 8.54	11 – 55	42.93 ± 8.61	11 – 55
**IHDI**	5	20.14 ± 5.59	5 – 25	20.48 ± 5.25	5 – 25	20.31 ± 5.42	5 – 25
**DHL**	4	15.00 ± 3.91	4 – 20	15.17 ± 4.12	4 – 20	15.08 ± 4.01	4 – 20
**MAAS**	13	44.87 ± 10.83	13 – 73	45.08 ± 12.11	13 – 78	44.97 ± 11.46	13 – 78
**HHPI**	14	59.91 ± 13.37	14 – 70	61.34 ± 12.40	14 – 70	60.60 ± 12.91	14 – 70

ASSHW: The Attitude Scale towards Social Handwashing on Nursing Students, IWI: Impact of Work Intensity, IHDI: Impact of Handwashing on Development of Infection, DLH: Desire to Learn Handwashing, MAAS: Mindful Attention Awareness Scale, HHPI: Hand Hygiene Practice Inventory.

### Relationship Between Descriptive Factors and Results of ASSHW and MAAS

Non-parametric tests were used to analyse differences in ASSHW and MAAS scores among student groups due to the non-normal distribution of the data. No significant group differences in ASSHW scores were observed based on the examined descriptive variables. However, regarding students’ place of residence, those who stayed at relatives’ homes had higher MAAS scores compared to other students.

### Correlational Analysis

Given the non-normal distribution of the data, Spearman’s rho correlation was applied to evaluate the relationships between students’ handwashing frequency, handwashing duration, and their scores on the ASSHW, MAAS, and HHPI scales. Significant positive correlations were found between the following variables (Table 4):

- IHDI and DLH (sub-dimensions of ASSHW) and MAAS, HHPI, handwashing frequency, and duration,
- DLH and MAAS, HHPI, handwashing frequency, and duration,
- MAAS and HHPI, handwashing frequency, and duration,
- HHPI and both handwashing frequency and duration,
- Handwashing frequency and duration themselves.

**Table 4:** Correlation test between students’ handwashing frequency and duration and scale scores – Mardin, Türkiye, 2024.

Scales		ASSHW	IWI	IHDI	DLH	MAAS	HHPI	Frequency of HH	Duration of HH
**ASSHW**	**r**	1.000							
	**p**	–––							
**IWI**	**r**	0.051	1.000						
	**p**	0.180	–––						
**IHDI**	**r**	–0.064	0.037	1.000					
	**p**	0.094	0.336	–––					
**DLH**	**r**	–0.034	–0.019	**0.392**	1.000				
	**p**	0.378	0.627	**0.000**	**––**				
**MAAS**	**r**	–0.036	–0.054	**0.297**	**0.655**	1.000			
	**p**	0.350	0.160	**0.000**	**0.000**	–––			
**HHPI**	**r**	–0.062	–0.002	**0.799**	**0.777**	**0.708**	1.000		
	**p**	0.108	0.954	**0.000**	**0.000**	**0.000**	–––		
**Frequency of HH**	**r**	–0.042	0.003	**0.196**	**0.294**	**0.296**	**0.331**	1.000	
	**p**	0.279	0.943	**0.000**	**0.000**	**0.000**	**0.000**	–––	
**Duration of HH**	**r**	–0.004	0.000	**0.459**	**0.532**	**0.482**	**0.592**	**0.223**	1.000
	**p**	0.927	0.993	**0.000**	**0.000**	**0.000**	**0.000**	**0.000**	–––

ASSHW: The Attitude Scale towards Social Handwashing on Nursing Students, IWI: Impact of Work Intensity, IHDI: Impact of Handwashing on Development of Infection, DLH: Desire to Learn Handwashing, MAAS: Mindful Attention Awareness Scale, HHPI: Hand Hygiene Practice Inventory.

These findings indicate that higher levels of mindful attention and awareness, as well as stronger attitudes toward hand hygiene, are positively associated with more frequent and prolonged handwashing behavior among students.

## DISCUSSION

This study examined the social handwashing attitudes and mindfulness levels of nursing and midwifery students, and the relationship between these variables. Findings confirmed H1, indicating that students generally held positive attitudes toward social handwashing and reported hand hygiene practices above a moderate level. These results are consistent with prior studies conducted among Turkish nursing students^(14,15)^.

Most students reported washing their hands when they felt their hands were dirty or during routine activities. Hospital practice emerged as a key influence, suggesting that students are particularly mindful of contamination risks during patient contact. While previous research noted that students often skipped handwashing due to forgetfulness^(16,17)^, participants in this study cited external barriers such as lack of products, unsuitable environments, or skin irritation. These findings align with studies emphasizing the impact of institutional culture and environmental support on hand hygiene compliance^(18,19)^. Therefore, hospitals and training institutions must ensure not only the availability of hygiene materials but also a supportive environment with positive role models to reinforce proper hand hygiene.

Students’ mindful attention levels (Mean ± SD: 44.97 ± 11.46) were comparable to those reported in previous literature^(20,21,22,23,24)^. No significant differences were found between nursing and midwifery students. A notable finding was that students living with relatives had higher mindful attention scores than those in dormitories, possibly reflecting the influence of a more supportive living environment on daily awareness.

Regarding H3, partial support was found for the association between mindful attention and hand hygiene. While overall mindful attention awareness (MAAS) scores were not directly correlated with total handwashing attitude scores (ASSHW), significant associations were identified between mindfulness and specific sub-dimensions of handwashing attitudes, as well as actual behavior (frequency and duration). These results suggest that mindful attention may influence the quality and consistency of hygiene practices rather than general attitudes.

These findings are consistent with previous studies suggesting that mindfulness supports health-promoting behavior, including hygiene adherence^(6,25)^. They also support Gilmartin et al.’s^(2)^ proposal that hand hygiene can serve as both a mindfulness prompt and a target for improvement through reflective awareness.

Given the growing attention to mindfulness-based interventions (MBIs) in healthcare education^(26,27,28)^, these findings have practical implications. Incorporating mindfulness training into nursing curricula could enhance students’ self-awareness and intentional behavior, particularly in routine yet critical tasks like handwashing. Although the evidence on the effectiveness of MBIs remains mixed^(3,29,30)^, their integration into clinical education deserves further exploration. For example, using hand hygiene as a context for teaching mindfulness could improve both behavioral outcomes and reflective practice.

The reliance on self-report measures may introduce bias, and the single-university sample limits generalizability. Additionally, the cross-sectional design prevents causal interpretation. Future research should consider observational or longitudinal designs and multi-center samples to validate and expand on these findings.

## CONCLUSION

The present study utilized multiple scale surveys among a large sample of Turkish nursing and midwifery students, which could be generalisable to other experimental and survey studies. Our findings confirmed that these students generally held positive attitudes toward handwashing and engaged in frequent handwashing practices, particularly in clinical settings. The level of mindful attention detected among the participants align with prior research and suggest potential for improvement through targeted interventions.

While mindfulness was positively correlated with handwashing practices, such as frequency and duration, its association with attitudes toward social handwashing was less evident. These results highlight the complexity of behavioral and attitudinal factors influencing hygiene compliance in healthcare students.

To promote better hand hygiene practices, institutions should focus on ensuring access to adequate hygiene facilities and integrating mindfulness–based approaches. Incorporating these findings into nursing and midwifery education could inform the development of training programs and updated curricula that not only reinforce practical hand hygiene behaviors but also support psychological readiness and mindful awareness. These strategies could ultimately foster a stronger culture of hygiene and patient safety in both educational and clinical settings.

## DATA AVAILABILITY

The datasets generated during and/or analyzed during the current study are available from the corresponding author on reasonable request.

## References

[1] Kabat-Zinn J (2013). Full catastrophe living, revised edition: how to cope with stress, pain
and illness using mindfulness meditation..

[2] Gilmartin H, Saint S, Rogers M, Winter S, Snyder A, Quinn M (2018). Pilot randomised controlled trial to improve hand hygiene through
mindful moments. BMJ Qual Saf.

[3] Shapiro SL, Carlson LE, Astin JA, Freedman B (2006). Mechanisms of mindfulness. J Clin Psychol.

[4] Chen SH, Chen PJ, Lee CH, Wu YP, Ahorsu DK, Griffiths MD (2023). Perceived stress mediating the association between mindfulness
and resilience among registered nurses. Psychol Res Behav Manag.

[5] Ropi I, Lillo M, Malavasi M, Argentieri A, Barbieri A, Lou B (2024). The psychological implications of COVID-19 over the
eighteen-month time span following the virus breakout in
Italy. Front Psychol.

[6] Wen X, Rafaï I, Duchêne S, Willinger M (2022). Did mindful people do better during the COVID-19 pandemic?
Mindfulness is associated with well-being and compliance with prophylactic
measures. Int J Environ Res Public Health.

[7] Haliwa I, Lee J, Wilson J, Shook NJ (2020). Mindfulness and engagement in COVID-19 preventive
behavior. Prev Med Rep.

[8] Dossey B, Keegan L (2008). Holistic nursing: a handbook for practice..

[9] Gilmartin HM (2016). Use hand cleaning to prompt mindfulness in clinic. BMJ.

[10] Creswell JW, Creswell JD (2018). Research design: qualitative, quantitative, and mixed methods
approaches..

[11] Kalkan N, Karadag M (2021). A study of development an attitude scale towards social hand
washing on nursing students [Hemşirelik öğrencilerinin sosyal el yikamaya
yönelik tutumlari ölçeği geliştirme çalişmasi].. Adiyaman Univ J Health Sci..

[12] Brown KW, Ryan RM (2003). Mindful attention awareness scale..

[13] Ozyesil Z, Arslan C, Kesici S, Deniz ME (2011). The validity and reliability study of Mindful Attention Awareness
Scale.. Educ Sci..

[14] Celik S, Karahan E, Aydim A (2022). Evaluation of nursing students’ hand hygiene knowledge, belief
and practices [Hemşirelik Öğrencilerinin El Hijyeni Bilgi, İnanç ve
Uygulamalarinin Değerlendirilmesi].. Health Commun..

[15] Bülbül Maraş G, Kocaçal E (2024). Exploring determinants of hand hygiene among nursing students: a
theory of planned behavior approach. BMC Nurs.

[16] Dutta G, Singh TG, Kumar T (2020). Knowledge and practice of hand hygiene among undergraduate
students and junior doctors in the Regional Institute of Medical Sciences,
Imphal. J Family Med Prim Care.

[17] Singh G, Singh R (2021). The practice of hand hygiene among undergraduate medical
students. MGM J Med Sci.

[18] Gould D, Drey N (2013). Student nurses’ experiences of infection prevention and control
during clinical placements. Am J Infect Control.

[19] Zimmerman PA, Sladdin I, Shaban RZ, Gilbert J, Brown L (2020). Factors influencing hand hygiene practice of nursing students: a
descriptive, mixed-methods study. Nurse Educ Pract.

[20] Morvay-Sey K, Trpkovici M, Ács P, Paár D, Pálvölgyi A (2022). Psychological responses of Hungarian students during the first
wave of the COVID-19 pandemic. Int J Environ Res Public Health.

[21] Azak A (2018). Determination the level of mindfulness of nursing
students. J Educ Res Nurs.

[22] Uzdil N, Günaydin Y (2022). The effect of sense of coherence on mindful attention awareness
and academic self–efficacy in nursing students. Nurse Educ Pract.

[23] Costa DAV, Kogien M, Hartwig SV, Ferreira GE, Guimarães MKOR, Ribeiro MRR (2022). Dispositional mindfulness, emotional regulation and perceived
stress among nursing students. Rev Esc Enferm USP.

[24] Güllü A, Aloğlu N (2024). The relationship between conscious awareness and HPV knowledge
levels of midwife and nursing students [Ebelik ve Hemşirelik Öğrencilerinin
Bilinçli Farkindaliklari ve Bilinçli Farkindalik ile HPV Bilgi Seviyesi
Arasindaki İlişki].. YOBU Fac Health Sci J..

[25] van Doren N, Mahlobo CT, Galla BM, Colaianne BA, Hirshberg MJ, Inkelas KK (2023). The role of mindfulness and compassion in early adults’
subsequent mental health, coping and compliance with health guidelines
during the COVID-19 pandemic: a prospective longitudinal
study. Soc Personal Psychol Compass.

[26] Lomas T, Medina JC, Ivtzan I, Rupprecht S, Eiroa-Orosa FJ (2019). A systematic review and meta-analysis of the impact of
mindfulness-based interventions on the well-being of healthcare
professionals. Mindfulness.

[27] Scheepers RA, Emke H, Epstein RM, Lombarts KM (2020). The impact of mindfulness-based interventions on doctors’
well-being and performance: a systematic review. Med Educ.

[28] Chen X, Zhang B, Jin SX, Quan YX, Zhang XW, Cui XS (2021). The effects of mindfulness–based interventions on nursing
students: a meta-analysis. Nurse Educ Today.

[29] Kaisti I, Kulmala P, Hintsanen M, Hurtig T, Repo S, Paunio T (2024). The effects of mindfulness-based interventions in medical
students: a systematic review. Adv Health Sci Educ Theory Pract.

[30] Guendelman S, Medeiros S, Rampes H (2017). Mindfulness and emotion regulation: insights from
neurobiological, psychological, and clinical studies. Front Psychol.

