# Supplementary material for: Mindful attention awareness and attitudes toward social handwashing among nursing and midwifery students
Source: Rev Esc Enferm USP. 2025 Oct 3;59:e20250095. doi: 10.1590/1980-220X-REEUSP-2025-0095en (PMC12503123; doi:10.1590/1980-220X-REEUSP-2025-0095en)
Supplement: Translation

# Translation: Atención plena o consciente (Mindfulness) y actitudes hacia el lavado de manos social entre estudiantes de enfermería y partería

**DOI:** 10.1590/1980-220X-REEUSP-2025-0095es

**Authors:** Leyla Baran, Huri Öztürk, Feride Doğru Mungan

**Affiliations:** 1 Mardin Artuklu University, Faculty of Health Sciences, Nursing Department, Mardin, Türkiye.; 2 Izmir Democracy University, Vocational School of Health Services, Elderly Care Department, İzmir, Türkiye.; 3 Mardin Artuklu University, The Institute for Graduate Educational Studies, Health Administration, Mardin, Türkiye.

**Keywords:** Desinfección de las Manos, Atención Plena, Estudiantes de Enfermería

## Abstract

**Objetivo:**

Explorar la relación entre la conciencia plena de la atención y las actitudes hacia el lavado social de manos entre estudiantes de grado en enfermería y obstetricia.

**Método:**

Estudio transversal y correlacional realizado con 679 estudiantes de grado en enfermería y obstetricia. Los datos se recolectaron mediante dos escalas: (1) la Escala de Conciencia Plena de la Atención (ECPA) y (2) la Escala de Actitudes hacia el Lavado Social de Manos (EALSM). Además, los participantes proporcionaron información sociodemográfica y completaron un inventario de prácticas de lavado de manos. Las relaciones entre las puntuaciones se analizaron utilizando el coeficiente de correlación de Spearman.

**Resultados:**

Los niveles de conciencia plena (ECPA) mostraron un promedio de 44.97 ± 11.46, y las actitudes hacia el lavado social de manos (EALSM) un promedio de 78.32 ± 13.67. Aunque no se encontró una correlación directa entre las puntuaciones totales de ECPA y EALSM, se identificaron correlaciones positivas entre los niveles de conciencia plena y ciertas subdimensiones de la EALSM, así como con comportamientos específicos de lavado de manos, como la frecuencia y la duración.

**Conclusión:**

Estos hallazgos sugieren que la conciencia plena puede favorecer mejores prácticas de higiene de manos entre estudiantes de enfermería y obstetricia.

## INTRODUCCIÓN

La atención plena o consciente (Mindfulness) consiste en centrarse intencionadamente en el momento presente con una actitud abierta e imparcial^(1)^. Su función es evitar que las personas actúen de forma automática, reaccionando en base a pensamientos automáticos y no analizados, y fomentar en su lugar una conciencia consciente de las experiencias que se desarrollan^(2)^. Esta mayor conciencia permite a las personas detenerse, observar y elegir respuestas intencionadas en lugar de reacciones habituales^(3)^.

Numerosas investigaciones han demostrado que la atención consciente desempeña un papel protector en la salud mental: amortigua el estrés^(4)^, ayuda a sobrellevar la ansiedad y la depresión^(5)^ y contribuye al bienestar general^(6)^. Más allá de los beneficios psicológicos, la atención consciente también se ha relacionado con un mejor cumplimiento de las medidas de salud preventiva, como el distanciamiento social, durante la pandemia de COVID-19^(7)^, lo que sugiere que las personas conscientes son más propensas a adoptar comportamientos impulsados por una intención consciente. Sin embargo, esta asociación no siempre se ha extendido al lavado de manos, una práctica de higiene fundamental pero a menudo automatizada^(7,8)^. La higiene de manos se realiza con frecuencia en entornos clínicos, especialmente por parte de los profesionales de enfermería y partería, y es propensa a descuidos cuando falta la atención^(9)^. Sin embargo, ofrece una oportunidad única para integrar la atención plena: al reenfocar la atención durante el lavado de manos rutinario, las personas pueden incorporar un momento de conciencia intencional en su práctica diaria^(2)^.

Desde un punto de vista teórico, la atención plena fomenta el comportamiento intencional al interrumpir la automaticidad y mejorar la conciencia metacognitiva. Cuando se aplica a la higiene de las manos, esto significa que la conciencia de la atención plena puede aumentar la probabilidad de realizar el comportamiento de forma fiable, consciente y reflexiva. Además, la teoría de la enfermería enmarca la higiene de las manos como un acto de autocuidado que da forma a la cultura del lugar de trabajo, lo que pone de relieve el potencial de las intervenciones de atención plena para reforzar tanto los hábitos personales como las normas organizativas más amplias^(8)^.

Por lo tanto, este estudio tuvo como objetivo investigar la relación entre la atención plena y las actitudes hacia el lavado de manos entre los estudiantes de enfermería y partería. También se realizaron análisis comparativos para explorar las diferencias en la conciencia plena y los comportamientos de higiene de manos (frecuencia y duración) según el género, el programa académico y el tipo de alojamiento. Nuestra hipótesis fue la siguiente:

H_1_:
Los estudiantes de enfermería y partería tienen actitudes positivas hacia el lavado de manos social.
H_2_:
Los estudiantes de enfermería y partería demuestran altos niveles de atención y conciencia plena.
H_3_:
Los niveles de atención y conciencia plena de los estudiantes de enfermería y partería están asociados con sus actitudes hacia el lavado de manos en la sociedad.

## MÉTODO

### Diseño del Estudio

En línea con los principios de la investigación cuantitativa que exploran las asociaciones entre variables o conjuntos de puntuaciones, este estudio utilizó un diseño de investigación correlacional transversal para examinar la relación entre el comportamiento de higiene de manos y la conciencia plena^(10)^.

### Población

Se reclutó a todos los estudiantes de enfermería y partería, desde el primer año hasta el nivel de posgrado, de la Facultad de Ciencias de la Salud de la Universidad de Mardin Artuklu durante el año académico 2023–2024.

### Criterios de Selección

En lugar de predeterminar el tamaño de la muestra, se empleó un método de muestreo aleatorio simple para incluir datos de toda la población objetivo. Los criterios de elegibilidad incluían estar matriculado en los programas de licenciatura en enfermería y partería durante el año académico y comprender y completar voluntariamente los formularios de recolección de datos. Se excluyó a los estudiantes que no firmaron el consentimiento informado para participar.

### Definición de la Muestra

La población total de estudiantes de enfermería y partería de la universidad era de 771 (417 estudiantes de enfermería y 354 estudiantes de partería). De ellas, diez estudiantes estaban ausentes cuando se recolectaron los datos y 82 se negaron a participar, lo que resultó en una tasa de respuesta del 88 %. Por lo tanto, 679 estudiantes participaron en el estudio.

### Recolección de Datos

***Cuestionario de características demográficas:*** El cuestionario demográfico fue elaborado por los investigadores basándose en la bibliografía reciente. Incluía ítems como la edad, el sexo, el tipo de alojamiento y preguntas relacionadas con los hábitos de lavado de manos de los participantes.

***Escala de actitud hacia el lavado de manos social en estudiantes de enfermería (Attitude Scale towards Social HandWashing on Nursing Students - ASSHW):*** La ASSHW evalúa las actitudes de los estudiantes de enfermería hacia el lavado de manos^(11)^. Se obtuvo permiso de los autores para utilizar la escala.

La escala constaba de 20 ítems, con 9 afirmaciones redactadas de forma positiva y 11 de forma negativa, que se respondían en un formato Likert de 5 puntos. Las puntuaciones obtenidas en la escala oscilan entre 20 y 100, donde las puntuaciones más altas indican actitudes más positivas hacia el lavado de manos. Las subdimensiones de la escala y los rangos de puntuación correspondientes son los siguientes:

- La primera subdimensión, Impacto de la intensidad del trabajo (***Impact of Work Intensity*** – **IWI**), incluye los ítems 1–11, con puntuaciones posibles que van de 11 (más baja) a 55 (más alta).
- La segunda subdimensión, Impacto del lavado de manos en el desarrollo de infecciones (***Impact of Handwashing on Development of Infection – IHDI***), incluye los ítems 12 a 16, con puntuaciones posibles que van de 5 (más baja) a 25 (más alta).
- La tercera subdimensión, Deseo de aprender a lavarse las manos (***Desire to Learn Handwashing- DLH***), incluye los ítems 17 a 20, con puntuaciones posibles que van de 4 (más baja) a 20 (más alta).

***Escala de la Atención consciente o Plena (Mindful Attention Awareness Scale – MAAS):*** La MAAS evalúa las diferencias individuales en la atención plena midiendo la conciencia y la atención a las experiencias presentes en la vida cotidiana^(12)^. Consta de 15 ítems calificados en una escala Likert de 6 puntos (de «casi siempre» a «casi nunca»). Las puntuaciones más altas indican una mayor atención plena. La MAAS tiene una estructura de un solo factor, que da una puntuación total única. La consistencia interna de la escala fue de 0,82. La adaptación turca demostró cargas factoriales de los ítems entre 0,48 y 0,81, una fiabilidad alfa de Cronbach de 0,80 y una fiabilidad test-retest de 0,86^(13)^. Se obtuvo permiso para utilizar la versión turca de la escala.

***Inventario de prácticas de higiene de manos (Hand Hygiene Practice Inventory – HHP):*** El HHP es un cuestionario de autoinforme diseñado para evaluar cuándo los estudiantes perciben la necesidad de lavarse las manos. Consta de breves ítems calificados en una escala Likert de 5 puntos.

### Análisis de Datos

Los datos se analizaron utilizando el paquete estadístico para ciencias sociales (SPSS) versión 26,0. Se utilizaron estadísticas descriptivas (medias, desviaciones estándar y porcentajes) para resumir las características demográficas y las puntuaciones de la escala.

La normalidad de los datos se evaluó mediante las pruebas de Kolmogorov-Smirnov y Shapiro-Wilk. Los resultados indicaron que todas las variables, incluida la frecuencia diaria de lavado de manos, la duración del lavado de manos y las puntuaciones de las escalas (ASSHW, IWI, IHDI, DLH, MAAS, HHPI), se desviaban significativamente de la distribución normal (p < 0,001). Por lo tanto, se emplearon pruebas estadísticas no paramétricas para las comparaciones entre grupos y los análisis de correlación.

Se examinaron las asociaciones entre las escalas, así como entre las puntuaciones de las escalas y la frecuencia y duración del lavado de manos, utilizando el coeficiente de correlación rho de Spearman. Se utilizó un intervalo de confianza del 95 % y se estableció un nivel de significación de p < 0,05 para todos los análisis.

### Aspectos Éticos

Este estudio fue aprobado por el Comité de Ética en Investigación (número de referencia: 2024/5-14).. Todos los procedimientos se ajustaron a los principios establecidos en la Declaración de Helsinki. Antes de comenzar la recolección de datos, se explicó el propósito del estudio a todos los participantes y se obtuvo su consentimiento informado por escrito.

### Disponibilidad de Los Datos

Los conjuntos de datos utilizados y analizados durante este estudio pueden solicitarse al autor correspondiente, siempre que la solicitud sea razonable.

## RESULTADOS

### Datos Descriptivos

La edad media de los participantes fue de 21,78 ± 1,84 años (rango de 18 a 30 años). La mayoría de los estudiantes eran mujeres (85,9 %), más de la mitad (51,25 %) eran estudiantes de enfermería, el 29,6 % eran estudiantes de cuarto año y el 75 % residían en residencias universitarias (Tabla 1).

**Tabla 1:** Características sociodemográficas de los participantes, Mardin, Turquía, 2024.

Características sociodemográficas		Enfermería		Partería		Total	
		n	%	n	%	n	%
**Género**	**Femenino**	252	72,4	331	100,0	583	85,9
	**Masculino**	96	27,6	–––	–––	96	14,1
**Años de estudio**	**1 año**	71	20,4	68	20,5	139	20,5
	**2o año**	84	24,1	75	22,7	159	23,4
	**3o año**	91	26,1	89	26,9	180	26,5
	**4o año**	102	29,3	99	29,9	201	29,6
**Estatus de vivienda**	**Casa de familia**	53	15,2	43	13,0	96	14,1
	**Casa de parientes**	4	1,1	2	0,6	6	0,9
	**Dormitorio colectivo**	236	67,8	273	82,5	509	75,0
	**Casa estudiantil**	55	15,8	13	3,9	68	10,0
**Total**		**348**	**100,0**	**331**	**100,0**	**679**	**100**

Como se muestra en la Tabla 2, la frecuencia media de lavado de manos fue de 12,70 ± 7,77 veces al día (rango: 1–50). Los participantes se lavaban las manos con mayor frecuencia cuando las sentían sucias (89,2 %) y preferían utilizar agua y jabón líquido (90,9 %) para la higiene de las manos. La duración media del lavado de manos fue de 34,59 ± 21,14 segundos (rango: 10–120 segundos).

**Tabla 2:** Distribución de los hábitos de lavado de manos de los estudiantes – Mardin, Turquía, 2024.

Hábitos de lavado de manos de los estudiantes	Enfermería		partería		Total	
	n	%	n	%	n	%
**Situaciones en las que los alumnos sienten la necesidad de lavarse las manos**						
Antes de usar el baño	186	53,4	161	48,6	347	51,1
Después de usar el baño	287	82,5	289	87,3	576	84,8
Después de regresar a casa	274	78,7	259	78,2	533	78,5
Antes de comer	265	76,1	232	70,1	497	73,2
Después de comer	271	77,9	263	79,5	534	78,6
Siempre que sienta las manos sucias	316	90,8	290	87,6	606	89,2
Antes de las prácticas en el hospital	214	61,5	193	58,3	407	59,9
Después de las prácticas en el hospital	280	80,5	268	81,0	548	80,7
**Materiales preferidos por los estudiantes para la higiene de las manos**						
Solo agua	37	10,6	19	5,7	56	8,2
Agua y jabón en barra	98	28,2	87	26,3	185	27,2
Agua y jabón líquido	316	90,8	301	90,9	617	90,9
Antiséptico a base de alcohol	141	40,5	103	31,1	244	35,9
**Frecuencia con la que se olvida lavarse las manos durante el día**						
Siempre	16	4,6	14	4,2	30	4,4
A menudo	14	4,0	22	6,6	36	5,3
A veces	75	21,6	57	17,2	132	19,4
Rara vez	170	48,9	156	47,1	326	48,0
Nunca	73	21,0	82	24,8	155	22,8
**Situaciones que afectan la frecuencia con la que los estudiantes se lavan las manos**						
Pasar el día en casa/al aire libre	288	82,8	262	79,2	550	81,0
Participar en prácticas hospitalarias	276	79,3	239	72,2	515	75,8
Tener un problema de salud	192	55,2	183	55,3	375	55,2
Estar en ambientes concurridos	223	64,1	214	64,7	437	64,4
Realizar tareas mecánicas sucias	226	64,9	209	63,1	435	64,1
Usar transporte público	235	67,5	210	63,4	445	65,5
**Situaciones que impiden a los alumnos lavarse las manos durante el día**						
Falta de acceso a productos de higiene	137	39,4	131	39,6	268	39,5
Irritación de la piel, grietas o reacciones alérgicas causadas por los productos para lavarse las manos	109	31,3	102	30,8	211	31,1
Dudas sobre la limpieza de los productos para lavarse las manos	108	31,0	96	29,0	204	30,0
Instalaciones inadecuadas para lavarse las manos	177	50,9	162	48,9	339	49,9
Ausencia de toallas de papel	95	27,3	78	23,6	173	25,5
Agendas apretadas o falta de tiempo	112	32,2	117	35,3	229	33,7
Preferencia por usar soluciones antisépticas en lugar de lavarse las manos	47	13,5	38	11,5	85	12,5
**Opiniones de los estudiantes sobre el uso de técnicas adecuadas para lavarse las manos**						
Sí	91	26,1	100	30,2	191	28,1
Parcialmente	205	58,9	196	59,2	401	59,1
No	37	10,6	28	8,5	65	9,6
No tengo opinión	15	4,3	7	2,1	22	3,2

Los factores que más influyeron en la frecuencia del lavado de manos fueron el tiempo que pasaban en casa o fuera de ella (81,0 %) y la participación en prácticas hospitalarias (75,8 %). Los cambios en la duración del lavado de manos se vieron influidos principalmente por el grado de suciedad de las manos (47,1 %) y la urgencia (22,2 %). Las principales barreras para el lavado de manos fueron la insuficiencia de las instalaciones de higiene (49,9 %), la falta de productos de higiene (39,5 %) y la irritación de la piel causada por los productos de limpieza (31,1 %).

La mayoría de los estudiantes (59,1 %) afirmaron cumplir parcialmente con las técnicas adecuadas de lavado de manos, mientras que el 28,1 % se mostraron seguros de su técnica.

Un análisis de la distribución de las puntuaciones de los estudiantes en la escala (Tabla 3) indicó que las puntuaciones medias fueron de 78,32 ± 13,67 para ASSHW, 42,93 ± 8,61 para el IWI, 20,31 ± 5,42 para el IHDI, 15,08 ± 4,01 para el DLH y 60,60 ± 12,91 para el HHPI, todas ellas por encima del nivel moderado. La puntuación media del MAAS fue de 44,97 ± 11,46 para ambos grupos de estudiantes.

**Tabla 3:** Análisis comparativo de ASSHW, MAAS y HHP – Mardin, Turquía, 2024.

Escalas	Número de ítems	Enfermería		Partería		Total	
		Media ± DE	Min – Max	Media ± DE	Min – Max	Media ± DE	Min – Max
**ASSHW**	20	78,19 ± 13,67	35 – 100	78,45 ± 13,68	45 – 100	78,32 ± 13,67	35 – 100
**IWI**	11	43,05 ± 8,69	11 – 55	42,80 ± 8,54	11 – 55	42,93 ± 8,61	11 – 55
**IHDI**	5	20,14 ± 5,59	5 – 25	20,48 ± 5,25	5 – 25	20,31 ± 5,42	5 – 25
**DHL**	4	15,00 ± 3,91	4 – 20	15,17 ± 4,12	4 – 20	15,08 ± 4,01	4 – 20
**MAAS**	13	44,87 ± 10,83	13 – 73	45,08 ± 12,11	13 – 78	44,97 ± 11,46	13 – 78
**HHPI**	14	59,91 ± 13,37	14 – 70	61,34 ± 12,40	14 – 70	60,60 ± 12,91	14 – 70

ASSHW: Escala de actitud hacia el lavado de manos social en estudiantes de enfermería, IWI: Impacto de la intensidad del trabajo, IHDI: Impacto del lavado de manos en el desarrollo de infecciones, DLH: Deseo de aprender a lavarse las manos, MAAS: Escala de atención consciente, HHPI: Inventario de prácticas de higiene de manos.

### Relación Entre Los Factores Descriptivos y Los Resultados del ASSHW y el MAAS

Se utilizaron pruebas no paramétricas para analizar las diferencias en las puntuaciones del ASSHW y el MAAS entre los grupos de estudiantes debido a la distribución no normal de los datos. No se observaron diferencias significativas entre los grupos en las puntuaciones ASSHW basadas en las variables descriptivas examinadas. Sin embargo, en cuanto al lugar de residencia de los estudiantes, los que se alojaban en casa de familiares obtuvieron puntuaciones MAAS más altas en comparación con los demás estudiantes.

### Análisis Correlacional

Dada la distribución no normal de los datos, se aplicó la correlación rho de Spearman para evaluar las relaciones entre la frecuencia con la que los alumnos se lavaban las manos, la duración del lavado y sus puntuaciones en las escalas ASSHW, MAAS y HHPI. Se encontraron correlaciones positivas significativas entre las siguientes variables (Tabla 4):

- IHDI y DLH (subdimensiones de ASSHW) y MAAS, HHPI, frecuencia y duración del lavado de manos.
- DLH y MAAS, HHPI, frecuencia y duración del lavado de manos.
- MAAS y HHPI, frecuencia y duración del lavado de manos.
- HHPI y frecuencia y duración del lavado de manos.
- Frecuencia y duración del lavado de manos por sí mismas.

**Tabla 4:** Prueba de correlación entre la frecuencia y la duración del lavado de manos de los estudiantes y las puntuaciones de la escala – Mardin, Turquía, 2024.

Escalas		ASSHW	IWI	IHDI	DLH	MAAS	HHPI	Frecuencia de lavado	Duración de lavado
**ASSHW**	**r**	1,000							
	**p**	–––							
**IWI**	**r**	0,051	1,000						
	**p**	0,180	–––						
**IHDI**	**r**	–0,064	0,037	1,000					
	**p**	0,094	0,336	–––					
**DLH**	**r**	–0,034	–0,019	**0,392**	1,000				
	**p**	0,378	0,627	**0,000**	**––**				
**MAAS**	**r**	–0,036	–0,054	**0,297**	**0,655**	1,000			
	**p**	0,350	0,160	**0,000**	**0,000**	–––			
**HHPI**	**r**	–0,062	–0,002	**0,799**	**0,777**	**0,708**	1,000		
	**p**	0,108	0,954	**0,000**	**0,000**	**0,000**	–––		
**Frecuencia de lavado**	**r**	–0,042	0,003	**0,196**	**0,294**	**0,296**	**0,331**	1,000	
	**p**	0,279	0,943	**0,000**	**0,000**	**0,000**	**0,000**	–––	
**Duración de lavado**	**r**	–0,004	0,000	**0,459**	**0,532**	**0,482**	**0,592**	**0,223**	1,000
	**p**	0,927	0,993	**0,000**	**0,000**	**0,000**	**0,000**	**0,000**	–––

ASSHW: Escala de actitud hacia el lavado de manos social en estudiantes de enfermería, IWI: Impacto de la intensidad del trabajo, IHDI: Impacto del lavado de manos en el desarrollo de infecciones, DLH: Deseo de aprender a lavarse las manos, MAAS: Escala de atención consciente, HHPI: Inventario de prácticas de higiene de manos.

Estos resultados indican que unos niveles más altos de atención y conciencia plena, así como unas actitudes más sólidas hacia la higiene de las manos, se asocian positivamente con un comportamiento de lavado de manos más frecuente y prolongado entre los estudiantes.

## DISCUSIÓN

Este estudio examinó las actitudes sociales hacia el lavado de manos y los niveles de atención plena de los estudiantes de enfermería y partería, así como la relación entre estas variables. Los resultados confirmaron la hipótesis H1, indicando que los estudiantes tenían en general actitudes positivas hacia el lavado de manos en el ámbito social y declararon prácticas de higiene de manos por encima de un nivel moderado. Estos resultados son coherentes con estudios previos realizados entre estudiantes de enfermería turcos^(14,15)^.

La mayoría de los estudiantes informaron que se lavaban las manos cuando sentían que estaban sucias o durante las actividades rutinarias. La práctica hospitalaria se reveló como una influencia clave, lo que sugiere que los estudiantes son especialmente conscientes de los riesgos de contaminación durante el contacto con los pacientes. Mientras que investigaciones anteriores señalaban que los estudiantes a menudo se saltaban el lavado de manos por olvido^(16,17)^, los participantes en este estudio citaron barreras externas como la falta de productos, entornos inadecuados o irritación de la piel. Estos hallazgos coinciden con estudios que enfatizan el impacto de la cultura institucional y el apoyo ambiental en el cumplimiento de la higiene de las manos^(18,19)^. Por lo tanto, los hospitales y las instituciones de formación deben garantizar no solo la disponibilidad de materiales de higiene, sino también un entorno propicio con modelos positivos que refuercen la higiene adecuada de las manos.

Los niveles de atención consciente de los estudiantes (media ± DE: 44,97 ± 11,46) fueron comparables a los descritos en la bibliografía previa^(20–24)^. No se encontraron diferencias significativas entre los estudiantes de enfermería y partería. Un hallazgo notable fue que los estudiantes que vivían con familiares tenían puntuaciones de atención consciente más altas que los que vivían en residencias, lo que posiblemente refleja la influencia de un entorno de vida más favorable en la conciencia diaria.

En cuanto a la hipótesis H3, se encontró un apoyo parcial a la asociación entre la atención consciente y la higiene de las manos. Si bien las puntuaciones generales de conciencia de la atención consciente (MAAS) no se correlacionaron directamente con las puntuaciones totales de actitud hacia el lavado de manos (ASSHW), se identificaron asociaciones significativas entre la atención plena y subdimensiones específicas de las actitudes hacia el lavado de manos, así como el comportamiento real (frecuencia y duración). Estos resultados sugieren que la atención consciente puede influir en la calidad y la consistencia de las prácticas de higiene más que en las actitudes generales.

Estos hallazgos son coherentes con estudios anteriores que sugieren que la atención plena favorece los comportamientos que promueven la salud, incluido el cumplimiento de las normas de higiene^(6,25)^. También respaldan la propuesta de Gilmartin et al.^(2)^ de que la higiene de las manos puede servir tanto como estímulo para la atención plena como objetivo de mejora a través de la conciencia reflexiva.

Dada la creciente atención que se presta a las intervenciones basadas en la atención plena (Mindful-Based Interventions - MBIs) en la educación sanitaria^(26-28)^, estos hallazgos tienen implicaciones prácticas. La incorporación del entrenamiento en mindfulness en los planes de estudios de enfermería podría mejorar la autoconciencia y el comportamiento intencional de los estudiantes, especialmente en tareas rutinarias pero críticas como el lavado de manos. Aunque las pruebas sobre la eficacia de las MBI siguen siendo contradictorias^(3,29,30)^, su integración en la educación clínica merece ser explorada más a fondo. Por ejemplo, el uso de la higiene de manos como contexto para enseñar mindfulness podría mejorar tanto los resultados conductuales como la práctica reflexiva.

La dependencia de medidas autoinformadas puede introducir sesgos, y la muestra de una sola universidad limita la generalización. Además, el diseño transversal impide la interpretación causal. Las investigaciones futuras deberían considerar diseños observacionales o longitudinales y muestras multicéntricas para validar y ampliar estos hallazgos.

## CONCLUSIÓN

El presente estudio utilizó encuestas de escala múltiple entre una amplia muestra de estudiantes de enfermería y partería turcos, que podrían generalizarse a otros estudios experimentales y de encuesta.

Nuestros hallazgos confirmaron que estos estudiantes tenían en general actitudes positivas hacia el lavado de manos y practicaban con frecuencia el lavado de manos, especialmente en entornos clínicos. El nivel de atención consciente detectado entre los participantes coincide con investigaciones anteriores y sugiere un potencial de mejora mediante intervenciones específicas.

Si bien la atención plena se correlacionó positivamente con las prácticas de lavado de manos, como la frecuencia y la duración, su asociación con las actitudes hacia el lavado de manos en el ámbito social fue menos evidente. Estos resultados ponen de relieve la complejidad de los factores conductuales y actitudinales que influyen en el cumplimiento de las normas de higiene entre los estudiantes de ciencias de la salud.

Para promover mejores prácticas de higiene de las manos, las instituciones deben centrarse en garantizar el acceso a instalaciones de higiene adecuadas e integrar enfoques basados en la atención plena. La incorporación de estos hallazgos a la formación en enfermería y partería podría servir de base para el desarrollo de programas de capacitación y planes de estudio actualizados que no solo refuercen los comportamientos prácticos de higiene de las manos, sino que también apoyen la preparación psicológica y la conciencia plena. En última instancia, estas estrategias podrían fomentar una cultura más sólida de higiene y seguridad del paciente tanto en entornos educativos como clínicos.

## DISPONIBILIDAD DE DATOS

Los conjuntos de datos generados y/o analizados durante el presente estudio están disponibles a través del autor de correspondencia previa solicitud razonable.
